# Classroom temperatures in urban and rural classrooms: disparities that hinder a just energy transition and opportunities for interventions

**DOI:** 10.11604/pamj.2026.53.98.49674

**Published:** 2026-02-25

**Authors:** Natasha Naidoo, Tracey Laban, Thandi Kapwata, Matthew Benyon, Caradee Yael Wright

**Affiliations:** 1 Environment and Health Research Unit, South African Medical Research Council, Pretoria 0001, South Africa,; 2 Environment and Health Research Unit, South African Medical Research Council, Johannesburg 2094, South Africa,; 3 Environmental Health Department, Faculty of Health Sciences, University of Johannesburg, Johannesburg 2094, South Africa,; 4 School of Physics and Astronomy, University of Leicester, Leicester, United Kingdom,; 5 Centre for Environmental Health and Sustainability, University of Leicester, Leicester, United Kingdom,; 6 Institute for Environmental Futures, University of Leicester, Leicester, United Kingdom

**Keywords:** Climate change, children, education, environmental health, heat, humidity, green building, school, thermal comfort

## Abstract

**Introduction:**

climate change is expected to raise ambient temperatures, with increases in Africa projected to exceed the global average. These rising temperatures impact indoor environments, including classrooms. This comparative observational field study aimed to compare classroom temperatures in urban and rural South African schools.

**Methods:**

indoor classroom temperatures were measured using iButtons in classrooms with varying building characteristics. Data on building characteristics, such as ceiling conditions, tree shading, and the presence of fans, were collected using a standardised observation checklist.

**Results:**

the maximum daily average temperature in the urban school was 32°C, while rural schools experienced temperatures as high as 42°C. Indoor temperatures and humidity levels at rural schools exhibited greater variability than those in urban classrooms, largely influenced by outdoor temperature conditions (p<0.001). This suggests that urban classrooms were better insulated. Fan cooling was effective in reducing temperatures in urban classrooms (p<0.001) but had no significant impact in rural classrooms (p=0.16). Broken or missing ceilings in rural schools contributed to higher temperatures (p<0.001). The study demonstrated that urban classrooms were better at maintaining the standard thermal comfort range of 25-28°C compared to rural classrooms.

**Conclusion:**

the findings reveal urban-rural inequalities in classroom thermal comfort due to poorer building infrastructure and limited effectiveness of low-energy cooling options. The disparities highlight a justice challenge within the energy transition, underscoring the need for equitable, climate-appropriate school infrastructure investments in South Africa, to ensure that adaptation benefits reach under-resourced rural communities. As South Africa moves toward a just energy transition economy, better building designs in school classrooms are a priority.

## Introduction

Environmental health risks are a growing concern for children, especially with the effects of climate change and the growing threat of rising global temperatures [1]. The Sustainable Development Goal 4 on quality education necessitates healthy school environments that are conducive to learning [2]. Thus, it is imperative that classrooms are suitable for optimal student health and performance. Several studies have analysed the effects of heat on health in children [3-7]. However, few studies examine classroom temperatures as a health risk factor for children in lower socio-economic areas in Africa, including South Africa.

Inadequate thermal comfort of children in South African classrooms has been linked to absenteeism and health effects such as tiredness, low concentration, headaches, and nausea [8-11]. Similar effects on health, learning, and academic achievement have been observed in classrooms across the world [3,12]. Children are especially sensitive to environmental stress because their bodies and minds are still growing. Their brain, nervous system, immune system, ability to regulate body temperature, and other important functions are developing during this time [13,14].

Numerous studies highlight the thermal discomfort and health consequences children experience in classrooms due to high temperatures. In India's humid subtropical climate, Grade 3 children were more sensitive to higher temperatures than Grade 5 children at 28-35°C [15]. In Yaoundé, Cameroon, the majority of students (48%) felt extremely hot (>32°C), fatigued (76%), and had headaches (38%) [16]. Headaches were more common in the afternoon, when temperatures were at their highest (32°C-36°C) [16]. During peak temperatures, researchers also noted absentmindedness (62%) and slower writing speed (45%) [16]. Hence, thermal comfort appears to be important for productivity and health, and well-being in the classroom. In Nigeria, students prefer short-walled open-plan buildings with natural ventilation over enclosed classrooms [17]. Open-plan classrooms are deemed necessary to save on air-conditioning [18], which is expensive and energy-intensive.

In South Africa, temperatures have increased by ±0.02°C annually [19,20] and high temperatures are expected to disproportionately affect the country's poorest neighbourhoods. This is particularly evident in South Africa, where, in lower-income communities, there is a noticeable absence of infrastructure and development of buildings where people live, work, and attend school. In addition, there is no legislation prescribed for public schools to address indoor thermal comfort standards. The only thermal comfort standards available are from the South African Labor Guide and the American Society of Heating, Refrigerating, and Air-Conditioning Engineers (ASHRAE) standards [21,22]. These standards are applicable to the climatic conditions of continents in the northern hemisphere, such as America and Canada. In addition, the standards do not consider the unique thermal comfort needs of children who tend to feel warmer than adults and are less likely to efficiently adapt to higher temperatures [23].

The conditions of schools in South Africa depend on the area in which the school resides. In the context of the South African education system, quintiles are used to rank public schools based on the socioeconomic status of the area in which they are located. Quintiles 1 to 3 denote schools that are in the most economically disadvantaged areas [24], also known as rural areas. Historically, these schools have been less developed than those in affluent quintiles 4 and 5 [24], which are typically located in urban areas. Schools in the low-income, rural district of Giyani recently brought the national government to task because school buildings were significantly broken-down, with flooring and ceilings falling apart and ceilings shattered owing to storms [25]. Thus, it is apparent that in South Africa, there are areas where enclosed classrooms lack wall and ceiling insulation, and other materials that maintain consistently lower indoor temperatures [10].

Given that there are schools in South Africa that do not have the infrastructure to support classroom thermal comfort, the goal of this study was to examine heat vulnerability in rural schools by investigating temperature disparities between urban and rural school classrooms in South Africa. The research objectives were: 1) to measure indoor temperatures in classrooms in an urban and rural setting; and 2) to investigate the temperature differences between these classrooms. The findings could assist policymakers and public administrators in developing strategies to address the challenge of health consequences from thermal discomfort, directing resources to the low socioeconomic areas that are most in need of improvements to building design and insulation.

## Methods

**Study design:** this study employed a comparative observational field design to assess indoor classroom temperature conditions in rural (Giyani) and urban (City of Tshwane) schools. Classroom temperature data loggers (iButtons) were installed in selected classrooms in rural schools and an urban school. Meteorological data from nearby weather stations were used to contextualize indoor measurements and assess the relationship between outdoor and indoor temperatures. The primary exposure variable was indoor classroom temperature, with rural and urban locations as the main predictors. Effect modifiers include seasonal variance and classroom building structural characteristics, which were included in this study. The study size was determined pragmatically based on the number of schools and classrooms that could be feasibly monitored within the study period, available data loggers, and logistical constraints.

**Figure 1:**
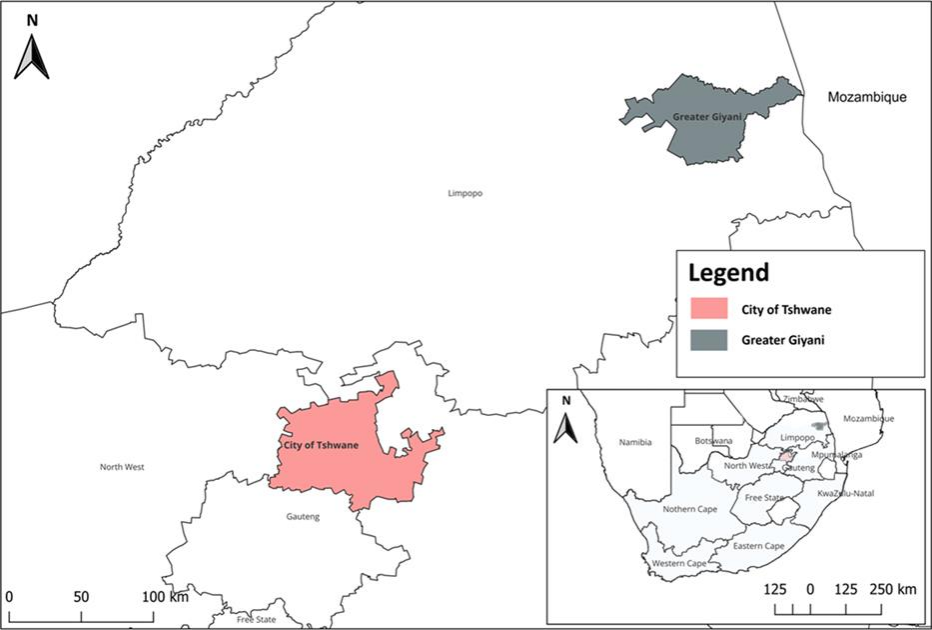
the study sites of the City of Tshwane and Greater Giyani

**Study setting:** this study included two types of locations in South Africa, namely urban and rural areas. The climate profiles of the City of Tshwane (urban setting) and the district of Giyani (rural setting) differ in that Tshwane is inland and landlocked, and Giyani is a coastal rural town. The City of Tshwane (Pretoria) (25.6051° S, 28.3929° E) has a population of 3,555,741 people (Figure 1) [26]. Pretoria experiences seasonal temperature variations with summer mean temperatures ranging from 17°C to 29°C during December, January, and February, with spring temperatures ranging from 24°C to 27°C, rarely falling below 18°C, during September, October, and November, and winter mean temperatures ranging from 2°C to 21°C during June, July, and August. Summers are long, warm, and partly cloudy, while winters are cold, dry, and clear. Throughout the year, mean temperatures typically vary from 6°C to 29°C, rarely falling below 2°C or rising above 32°C [27].

The rural schools were situated in the Giyani Local Municipality, which is a part of the Mopani District Municipality in Limpopo (23.3127° S, 30.7034° E) (Figure 1). Giyani local municipality has a population of 247,657 inhabitants in 57,537 households, mainly clustered in hamlets or villages [28]. Giyani experiences long, hot, humid, and partly cloudy summers, and short, cool, dry, and clear winters. Annual mean temperatures vary from 10°C to 30°C, rarely dropping below 7°C or exceeding 36°C [27]. During summer (December-February), average daytime high temperatures range from 28°C to 31°C, with overnight lows of 19-20°C, indicating persistently warm conditions even at night [27]. Spring (September-November) is similarly warm, with average highs of 28-29°C and lows of 16-18°C, while autumn (March-May) sees slightly milder conditions, with average highs between 25°C and 28°C and lows of 14-18°C [27]. Winters in Giyani (June-August) remain relatively warm compared to many other regions, with average daytime highs of 22-25°C and nighttime lows of 11-13°C [27].

The rural site was specifically chosen for its low socioeconomic profile. The average annual household income in the district is R14,600 (842.36 US dollars as of the 1^st^ Oct 2024), which is nearly half the national average of R29,400 (1,696.20 US dollars as of the 1^st^ Oct 2024) [29]. Additionally, the Greater Giyani Local Municipality has the highest percentage of people living in poverty, with a total of 77%. The area's significant socioeconomic challenges are a focus for this study.

**Temperature and relative humidity measurements:** classrooms were purposively sampled to capture realistic variations in building characteristics known to influence indoor thermal conditions, while reflecting typical infrastructure in urban and rural public schools. Temperature data were collected across autumn (Mar-May), winter (Jun-Aug), and spring (Sep-Nov). Monitoring took place from 14 Apr-30 Sep 2023 for the urban school and 26 Apr-12 Oct 2022 for the rural schools, except School 4 (Giyani), which was monitored from 31 May-12 Oct 2022. Permission was obtained from school management, and no students participated. Temperatures were recorded every 5 minutes using Thermochron iButton® DS1923 loggers. In both urban and rural settings, six classrooms per school were equipped with two loggers each for backup. Devices were securely mounted about 2 m high on cupboards or walls, away from direct sunlight and open windows, ensuring reliable and consistent measurements.

**Classroom building characteristics:** at baseline data collection, during logger setup, fieldworkers used a standardised checklist to record classroom building characteristics, including construction materials, ceiling presence, presence of functional cooling measures, and external features such as tree shade. Data were captured on paper forms, entered into Excel, and included in the statistical analysis.

**Temperature data:** outdoor temperature and relative humidity values were collected from the South African Weather Service. Temperature data for the iButtons for school weekdays and school hours starting at 8h00 and ending at 14h00 (local school hours). The 5-minute readings were averaged into hourly records, and boxplots were constructed to display daily temperature variation during school hours for each classroom.

**Apparent temperature calculations:** apparent temperature (AT) was calculated from the measured indoor temperature and relative humidity readings from the iButtons. AT is a measure of how people experience or perceive temperature [30]. Apparent temperature (AT) was calculated as an adjusted ambient temperature depending on the relative humidity level, wind speed, and elevation [30]. The AT was calculated using the equation [30,31]:

AT=T_α_+ 0.33 ×e (-0.70) × ws-4.00

Where: Tα = dry bulb temperature (°C); e = water vapour pressure (hPa); ws = wind speed (m/s) (set to 0 because this was an indoor setting).

*e* = *rh*/100 × exp (17.27 × *Tα*/(237.7 + *Tα*))

Where: rh = relative humidity (%); exp = exponential function (base e, Euler’s number ≈ 2.71828).

**Statistical analysis:** data were examined for missing values and temporal gaps before analysis. Missing data were minimal, and these were excluded from analysis. Boxplots were used to visualise daily temperature variation during school hours using RStudio. Sensitivity analysis included calculated indoor Ats, which were compared to an international symptom table developed by the United States National Weather Service (NWS) and the National Oceanic and Atmospheric Administration (NOAA) as previously described [30]. According to these standards, a “caution" warning is recommended at an AT range of 26.7-31.7°C [30]. An “extreme caution” warning is recommended at an AT range of 32.2-40°C [30].

Time series plots and simple linear regression were generated in R Studio to compare indoor and outdoor temperatures. Histograms (Annex 1) indicated non-normal distributions, so a Mann-Whitney U test was used to assess differences in indoor temperatures between urban and rural schools. To analyse the temperature variations between classrooms, the 5-minute AT values were averaged into hourly records. Line graphs were generated to compare the mean AT values across classrooms throughout the day (Microsoft Excel). Simple linear regression and Tukey analysis were conducted to evaluate the association between school building characteristics (such as fan cooling, tree shade, and intact ceilings) and AT within classrooms. Statistical analyses were done using R software, which is freely available online.

**Ethics consideration:** ethics clearance was granted by the South African Medical Research Council Research Ethics Committee (EC005-3/2014).

## Results

**Classroom descriptions:** in total, 15 classrooms were included in the study: 6 classrooms from the urban school in Tshwane (A-F) and 9 classrooms from six rural schools in Giyani (1A-6B). All classrooms had metal roofs and predominantly brick walls, ensuring baseline comparability across sites, while deliberate variation was retained in ceiling condition (intact, absent, or partially present), cooling methods (natural ventilation vs. fans), wall finishes, colour, and tree shading (Table 1).

**Table 1:** classroom characteristics of the school buildings

School/ Class	Wall type	Roof material	Exterior walls	Colour	Cooling methods	Ceiling boards	Tree Shading
**Urban school**							
A	Brick	Metal	Plastered	Beige	Natural	Intact	Absent
B	Brick	Metal	Plastered	Beige	Natural	Intact	Absent
C	Brick	Metal	Plastered	Beige	Natural	Intact	Absent
D	Brick	Metal	Plastered	Beige	Wall-mounted fan	Intact	Present
E	Brick	Metal	Plastered	Beige	Natural	Intact	Absent
F	Prefab asbestos	Metal	Plastered	Beige	Natural	Intact	Present
**Rural school**							
1/A	Brick	Metal	Plastered	Brown	Ceiling fan	Absent	Absent
1/B	Brick	Metal	Plastered	Brown	Ceiling fan	Absent	Absent
2/A	Brick	Metal	Plastered	White	Ceiling fan	Present#	Absent
2/B	Brick	Metal	Plastered	White	Ceiling fan	Present	Absent
3	Brick	Metal	Plastered	Beige top / green bottom	Natural	Present	Absent
4	Brick	Metal	Un-plastered	Brown	Ceiling fan	Intact	Absent
5	Brick	Metal	Plastered	Light beige	Natural	Absent	Absent
6/A	Brick	Metal	Plastered	Beige top / red bottom	Ceiling fan	Present	Absent
6/B	Brick	Metal	Plastered	Beige top / red bottom	Ceiling fan	Present	Absent

# All conductivity measurements are given in W/mK

The urban sample (n = 6 classrooms) represents relatively well-maintained infrastructure, with intact ceiling boards in all classrooms, limited use of mechanical cooling (one wall-mounted fan), and minimal tree shading, alongside one prefabricated asbestos classroom to reflect common urban school heterogeneity. In contrast, the rural sample (n = 9 classrooms) captures greater structural diversity and vulnerability, including absent or damaged ceilings, un-plastered walls, heavier reliance on ceiling fans, and a general absence of tree shading, which are characteristic of under-resourced rural schools.

**Figure 2:**
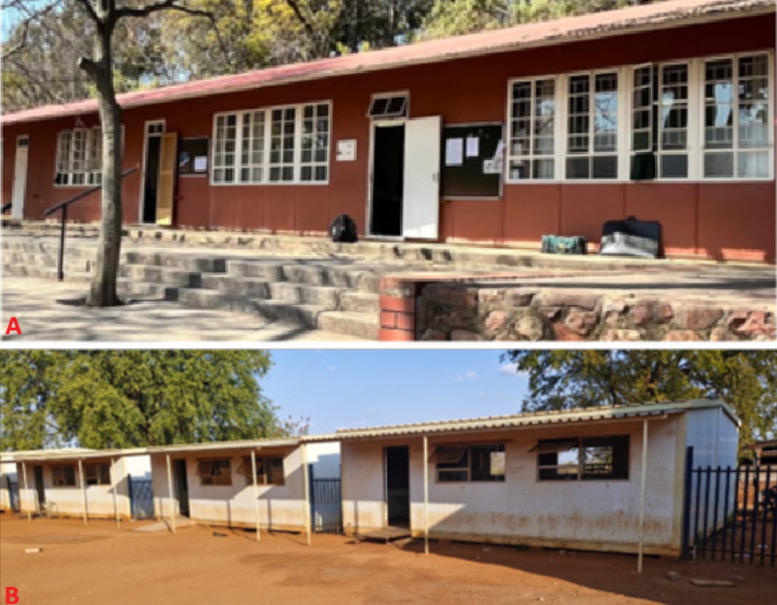
A) urban school - city of Tshwane, and B) rural school - one of the six rural schools in Giyani

Disparities in school infrastructure were observed and documented between urban and rural settings (Figure 2 A and B). The urban school showed more robust construction, potentially better spatial planning, and superior maintenance, whereas rural school buildings appeared less systematically designed and structurally less developed. These differences likely impact indoor thermal conditions and the overall learning environment. All classrooms studied were brick with plastered interior walls and corrugated iron (metal) roofs, except for one prefabricated asbestos classroom at the urban school. Both urban and rural schools had some classrooms equipped with ceiling or wall-mounted fans (Table 1). The urban school had a few classrooms with exterior trees providing shade. The rural school had a few classrooms with missing or damaged ceilings.

**Temperatures in classrooms:** in the urban school during winter, mean hourly temperatures ranged from a low of 14±2.8°C (classroom C) to a high of 17±3.0°C (classroom D), with maximums from 23°C (classroom B) to 27°C (classroom E) (Table 2). Autumn temperatures rose, spanning a mean of 20±4.1°C (classroom C) to 23±3.7°C (classroom A), with a maximum of 32°C (classroom F). Spring means were tighter, from 20±2.5°C (classroom C) to 23±3.0°C (classroom E), and a peak maximum of 30°C (classroom E) (Table 2). The rural school showed a wider temperature range. In winter, mean hourly temperatures varied from 19±2.5°C (class 6B, which also had the lowest minimum of 14°C) to 23±2.1°C (class 2A), and a maximum of 32°C (class 1A) (Table 2). Autumn saw class 1A record the highest mean of 27±4.4°C and maximum of 35°C, while class 6B had the lowest mean (22±2.4°C) and minimum (15°C). By spring, temperatures increased significantly: class 1B had the highest mean (29±4.4°C), class 1A reached the peak temperature of 41°C, and class 6B again had the lowest mean (25±3.6°C) (Table 2).

**Table 2:** hourly average indoor temperatures in urban and rural classrooms

Rural Schools					Urban School				
Class	Mean ± SD (°C)	Median (°C)	Minimum (°C)	Maximum (°C)	Class	Mean ± SD (°C)	Median (°C)	Min (°C)	Max (°C)
**Winter**									
1A	22 ± 4.5	23	13	32 #	A	17 ± 2.5	17	10	24
1B	22 ± 3.5	23	13	27	B	15 ± 2.8	15	7	23
2A	23 ± 2.0	23	18#	27	C	14 ± 2.8	13	7	24
2B	21 ± 2.2	21	17	25	D	17 ± 3.0	17	**9 #**	25
3	21 ± 2.1	21	17	25	E	17 ± 3.6	17	8	**27 #**
4	22 ± 3.1	22	16	28	F	17 ± 2.9	16	9	25
5	20 ± 2.6	20	14	25					
6A	22 ± 2.2	22	17	26					
6B	19 ± 2.5	19	14	24					
**Autumn**									
1A	27 ± 4.4	28	15	**35**	A	23 ± 3.7	22	15	31
1B	26 ± 3.1	27	16	32	B	21 ± 3.2	21	13	30
2A	26 ± 1.7	26	20	30	C	20 ± 4.1	20	12	29
2B	25 ± 2.0	26	19	29	D	22 ± 3.4	22	14	30
3	25 ± 1.8	26	20	31	E	22 ± 3.1	23	12	31
4	NA	NA	NA	NA	F	22 ± 3.4	22	13	32
5	24 ± 2.7	25	16	33					
6A	26 ± 2.6	27	15	32					
6B	22 ± 2.4	22	15	29					
**Spring**									
1A	29 ± 5.6	28	14	41	A	22 ± 2.2	22	17	27
1B	29 ± 4.4	29	15	39	B	22 ± 2.2	22	17	26
2A	28 ± 2.8	28	21	33	C	20 ± 2.5	20	14	26
2B	27 ± 3.9	27	19	35	D	22 ± 2.7	22	15	28
3	28 ± 3.3	28	19	34	E	23 ± 3.0	23	16	30
4	29 ± 3.8	30	19	37	F	22 ± 2.2	22	16	27
5	27 ± 4.3	27	17	38					
6A	28 ± 3.1	28	20	35					
6B	25 ± 3.6	24	16	34					

# Numbers in bold represent the highest temperature during the season

**Figure 3:**
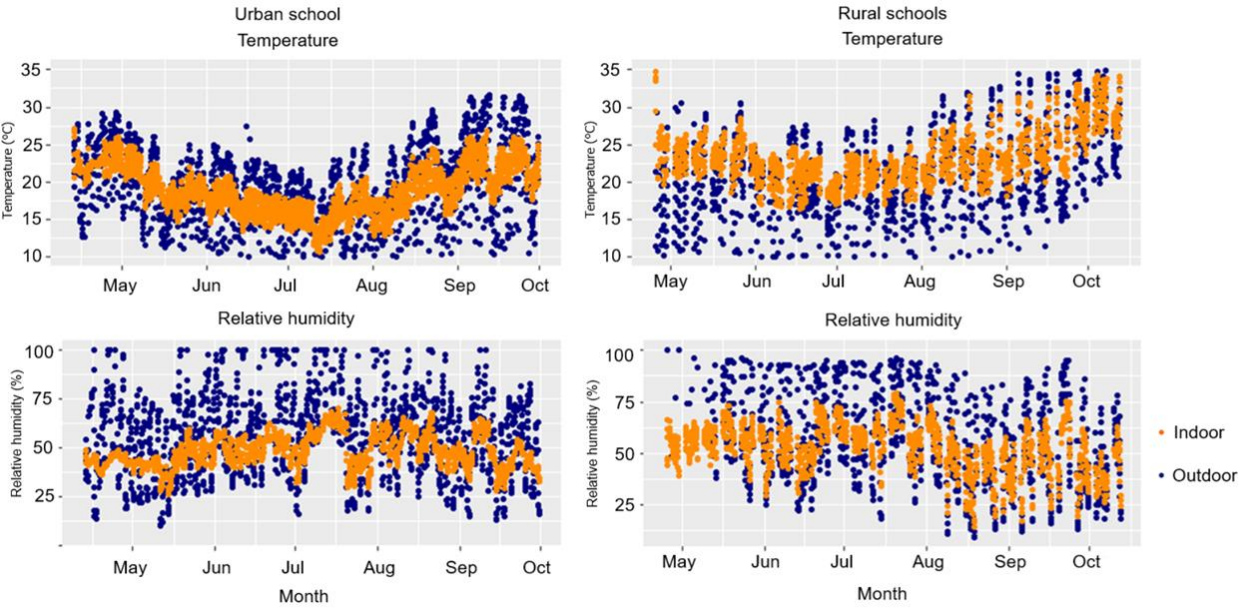
time series of hourly indoor (orange) and outdoor (blue) temperature (top) and relative humidity (bottom) in urban (left) and rural (right) classrooms

**Outdoor versus indoor temperatures:** Figure 3 compares indoor and outdoor temperature and relative humidity (RH) in urban and rural settings over the study period. Both temperature and RH followed seasonal trends, with cooler conditions in June-July and warmer, drier conditions in September-October. Outdoor values were generally more variable than indoor ones. Rural outdoor temperatures showed wider extremes (10°C-33°C) compared to the urban setting, where temperatures remained more stable and below 25°C until late August. Rural classrooms exhibited greater indoor temperature variation that closely mirrored outdoor conditions, suggesting limited insulation. They also had higher indoor RH that followed outdoor trends. In contrast, urban classrooms were less influenced by outdoor conditions, indicating better protection or construction quality. Although rural sites experienced higher outdoor temperatures overall, urban indoor temperatures remained below 27°C even when outdoor conditions reached the “Extreme Caution” threshold (32°C).

Linear regression analysis of outdoor temperature as a predictor of indoor temperature (outcome variable) showed that outdoor temperature was a significant predictor of indoor temperature at both urban (RE= 1.91, R2= 0.71) and rural (RE= 1.84, R2= 0.65) schools. Rural classroom temperatures were higher (intercept= 13.74) compared to the urban classrooms (intercept= 11.04). Rural classrooms' indoor temperatures were more sensitive to outdoor temperature changes (rural classrooms slope= 0.463 vs. urban classrooms slope= 0.423). In rural classrooms, a higher R2 indicated that outdoor temperatures were a stronger predictor of indoor temperatures. Rural classrooms are slightly more responsive to changes in outdoor conditions.

**Direct comparison of indoor temperatures in urban and rural schools:** the Mann-Whitney U test showed a statistically significant difference in temperatures between urban and rural schools (W = 294356, p < 0.05), indicating that the difference between the distributions of the urban and rural schools was likely not due to chance.

**Figure 4:**
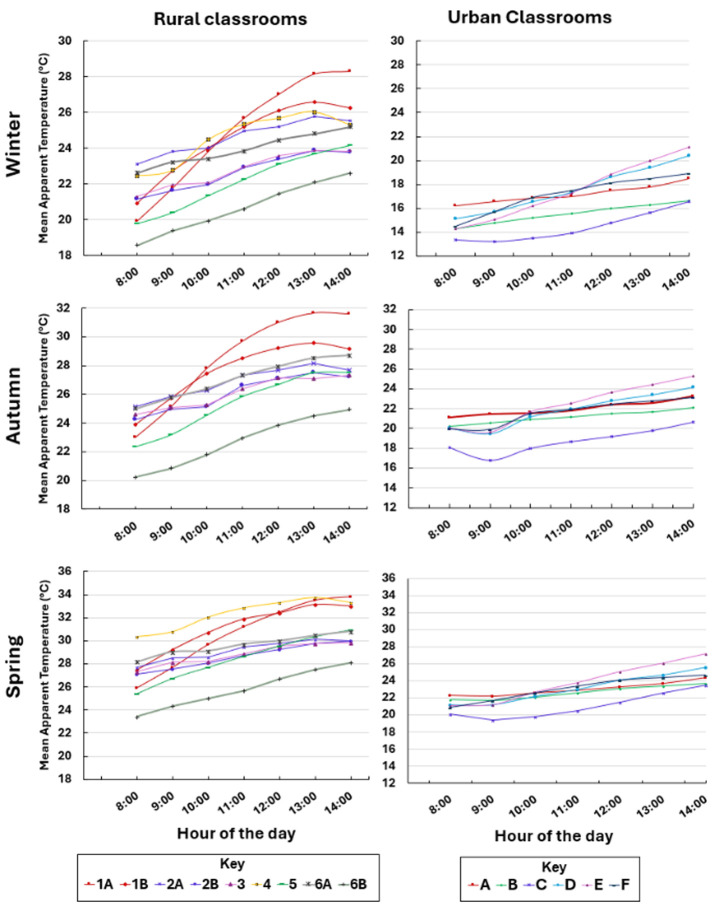
hourly indoor temperatures for rural and urban classrooms by season

**Figure 5:**
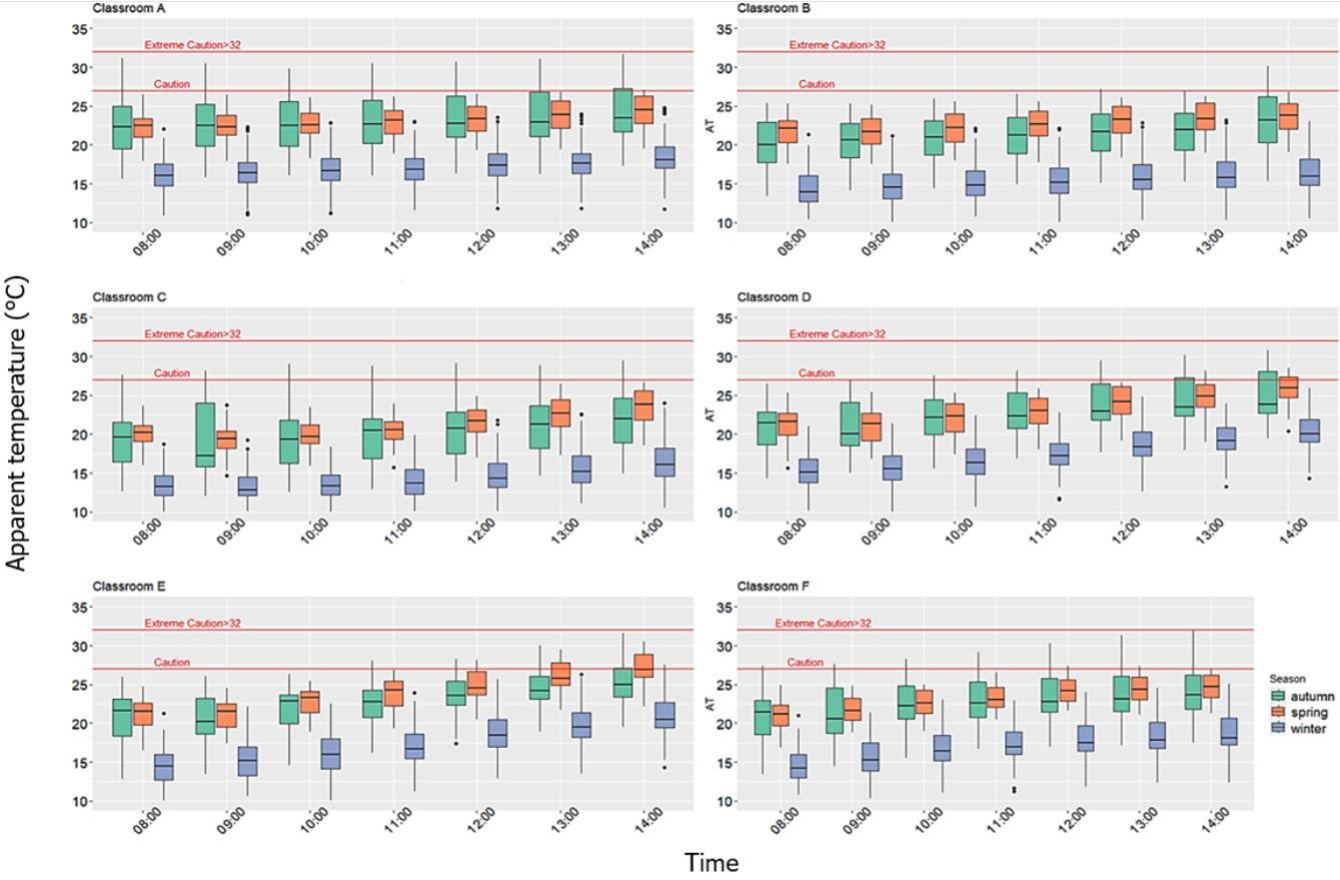
urban setting: indoor temperature distribution by classroom and time of day; box plots indicate the maximum (top of vertical line), 75^th^ percentile (top of box), median (horizontal line in box), 25^th^ percentile (bottom of box) and minimum (bottom of vertical line) temperatures

**Figure 6:**
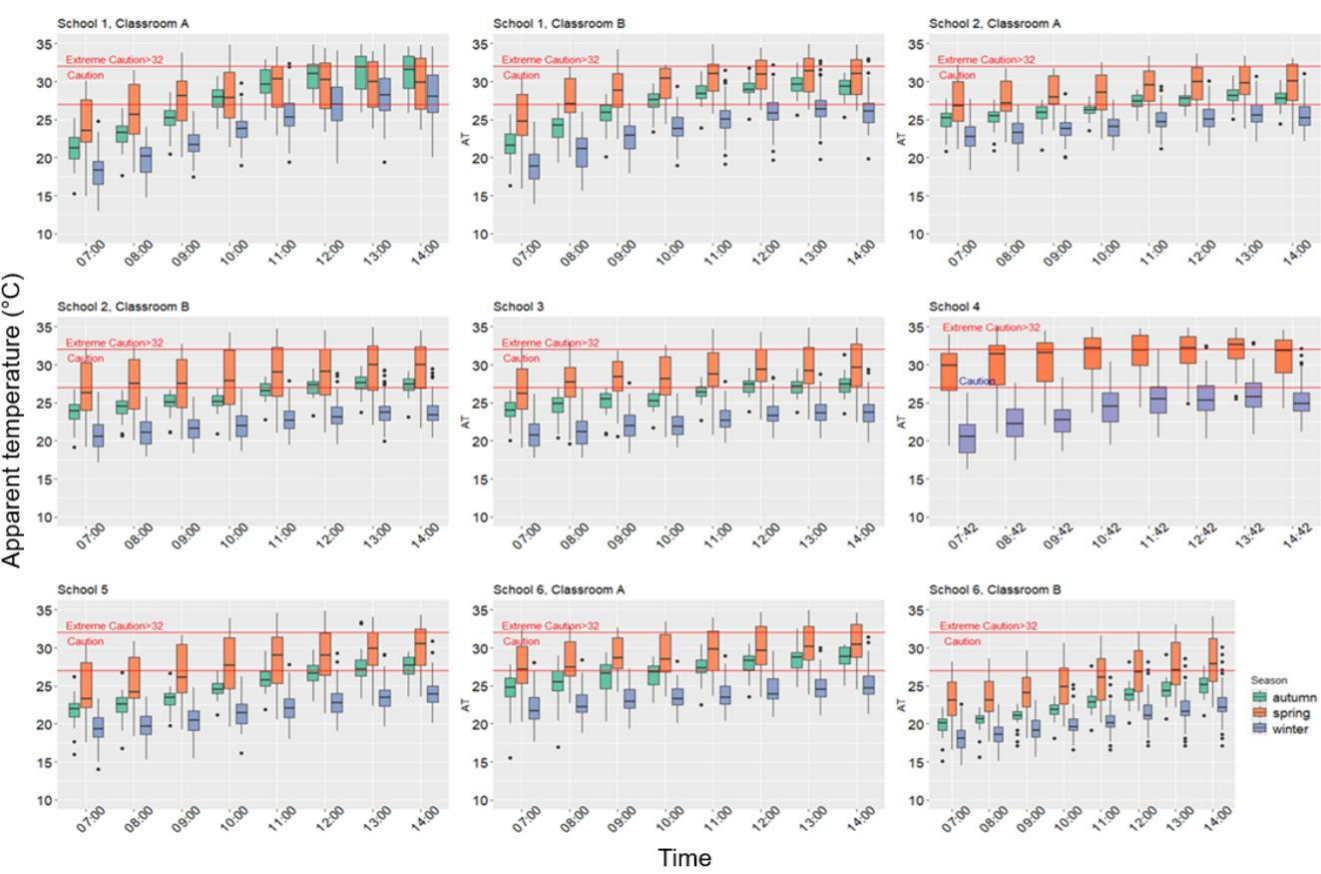
rural classrooms' temperature distribution by classroom and time of day; box plots indicate the maximum (top of vertical line), 75th percentile (top of box), median (horizontal line in box), 25th percentile (bottom of box) and minimum (bottom of vertical line) temperatures

**Trends in classroom temperatures:** Figure 4 shows diurnal classroom temperature patterns by season for urban and rural schools. Rural classrooms generally reached higher temperatures and showed greater variation between classrooms than urban ones. The least variation occurred in the urban setting during spring. Differences in rural classrooms likely reflect variations between schools, though even within the same school (e.g., school 6), notable temperature differences were observed. Figure 5 shows diurnal indoor temperature boxplots for each classroom, with all except classroom E remaining within safe thermal comfort limits. In contrast, Figure 6 shows that most rural classrooms exceeded these limits. During winter, temperatures were generally safe except in school 1, classroom A, which often exceeded the “caution” threshold in the afternoons.

**Association of indoor temperatures with building characteristics:** to assess links between classroom characteristics and air temperature (AT), we combined checklist data with indoor temperature measurements (Table 3). Classrooms with fan cooling in urban schools showed significantly lower temperatures than those with natural ventilation, while fans had little effect in rural schools. Intact ceilings were associated with lower temperatures than broken or absent ceilings, though Tukey post-hoc tests showed overlapping confidence intervals, suggesting a larger sample may be needed for clearer significance. Tree shade had no significant effect on classroom temperatures.

**Table 3:** the linear regression analysis results for ATs at the urban and rural schools

Classroom characteristic comparisons	Linear regression	
	R2 value	p-value
**Urban school**		
Natural ventilation | Fan cooling	0.216	p < 0.05
Tree shading | no tree shading	0.004	0.19
**Rural school**		
Natural ventilation | Fan cooling	0.005	0.16
No ceiling | broken ceiling | intact ceiling#	0.104	p < 0.05

* True difference in means is not equal to 0 # Tukey model used to compare the ceiling categories after ANOVA

## Discussion

Our previous studies showed that classrooms with metal roofing were hotter than those with tiled roofs [9,10]. Despite the use of metal roofing, urban schools in this study generally maintained lower indoor temperatures on hot days and warmer temperatures on colder days than rural classrooms. A recent study of temporary classrooms (container and mobile) at affluent schools in the Western Cape (quintile 5) found that they have significantly poorer thermal comfort conditions, with temperatures closely mimicking outdoor temperature conditions significantly more than brick and prefabricated asbestos classrooms, even in the presence of an air conditioning unit [32]. This highlights the importance of considering insulation to maintain thermal comfort when designing school buildings.

Rural schools showed a propensity to exceed the NOAA “extreme caution” threshold (>32.2°C). The temperatures in the urban school classrooms, for the most part, stayed below the NOAA “caution” warning (26.7 - 31.7°C). This pattern is consistent with a recent study conducted by our group, which reported hourly mean temperatures of 34°C and humidity levels reaching a maximum of 80.4%, exceeding recommended thresholds set by the World Health Organization and the United States Environmental Protection Agency [33]. Of the 456 apparent temperature observations recorded, 203 (44.5%) were classified as posing no health risk, 226 (49.6%) fell within the “caution” category, and 27 (5.9%) were within the “extreme caution” category [33]. These higher apparent temperature ranges are associated with adverse health outcomes, including heat cramps, heat exhaustion, and, with prolonged exposure, heat stroke. Regression analyses indicated that for every 1°C increase in apparent temperature, a 0.05-unit increase in the number of learners reporting feelings of tiredness between 08: 00 and 09: 00 was predicted; however, this association was not statistically significant [33].

According to ASHRAE standards, the recommended temperature range for optimal indoor thermal comfort is 20°C to 26°C. The study calculated the adaptive thermal comfort of participants based on the adaptive thermal model, which suggests that occupants adapt to indoor and outdoor temperature changes over time [34]. Young students in the Philippines' Bicol region showed evidence of a decline in test scores at temperatures >25 °C, indicative of poor cognition in hot environments [35]. When temperatures were reduced from 25.3°C to 23.1°C, cognitive performance improved by approximately 6-8% [35]. Students in Australia expressed a preference for an acceptable summertime range from 19.5 to 26.68°C [36]. Young children in Chile preferred an adaptive thermal comfort level of 22.5°C to 23.1°C in the spring [34]. However, mean temperatures from 11: 00 am onwards exceeded this range in most classrooms except the classroom with air conditioning. Thus, there is an increased risk of thermal discomfort in classrooms without air-conditioning, which could impact learner concentration and productivity.

A point of concern in this and previous studies [8,10] is the existence of asbestos materials in prefabricated walls that have been used to build schools in the past. The prefabricated asbestos buildings pose a health risk to learners because the material is dangerous to human health as it is carcinogenic [37]. The South African Department of Education issued a mandate to reduce the number of school buildings made from asbestos, but measures have not been taken to remove several of these buildings [38]. As these buildings get older and there is a greater chance of leakage of asbestos [38], school principals need to report such cases, and these buildings should be maintained or replaced immediately. Unfortunately, the government has declared that there are insufficient funds to address the issue [38].

As rural classrooms in Giyani are more vulnerable to extremes in outdoor temperatures, investing in sustainable and resilient building designs is likely to protect young scholars from the health risks associated with both heat and cold stress. The key lesson is that building design in rural areas like Giyani needs better infrastructure to regulate indoor temperatures and maintain thermal comfort. School buildings in Giyani and other rural areas need to be designed to manage both hot and cold extremes due to greater temperature variations. These structures should include features like better insulation, shading, and ventilation to minimize the effect of external temperatures on indoor environments. This contrasts with Tshwane, where buildings already seem to maintain a more stable indoor climate. This points to the need for improved construction materials and techniques, such as insulated roofing, ceiling fans, and shaded windows. Buildings in Giyani could benefit from passive cooling strategies such as tree shading, reflective materials, or ventilation systems designed to cool without heavy energy consumption. Tshwane´s classrooms appear to already manage this to some extent, so transferring those strategies to Giyani might help address the thermal discomfort issue.

Thermal regulation in building design is more prominent in high-income countries (HICs). For example, the Réunion Islands (a province of France), in a tropical climate, have several net-zero energy buildings that combine mixed-mode air-conditioning, solar protection, and natural ventilation [18]. In this way, thermal comfort is achieved whilst excess air-conditioning is lowered [18]. Low- and middle-income countries (LMICs)s and low socioeconomic groups in HICs often lose out on the option of regulating temperatures in their classrooms. The challenge in LMICs is the high cost of air conditioning and reliable energy supplies. However, if students are not allowed to be comfortable in the classroom, their academic performance likely suffers.

It has been projected that the energy demand for cooling in HICs will rise by a range of 300-600% due to rising temperatures that have made heat in these regions unbearable [39]. Solar air conditioning was shown to be a feasible and economically viable option in a Baghdad sports arena [40]. The cooling load was 700 to 800 kW for a 2000 to 2500 m2 area, with costs estimated at 4-5 dollars per hour [40]. With up to 3 639 solar cooling publications on the Web of Science in the year 2020 [41], more action needs to be taken to make solar-powered air-conditioners accessible to developing countries, especially public/government schools in Africa.

An alternative to solar air conditioning in cases where there are very few funds available for school building design and construction is the concept of green building [42]. Green buildings emphasize the direction that buildings face by simulating the best orientation of buildings so that there is less sun and overheating exposure [42]. High-performance, low-emissivity glazed windows will minimize energy consumption for heating while reducing heat gain from the sun to reduce cooling needs, without limiting the quantity of light that enters a classroom [42]. A great method of insulation for external walls includes 200mm thick insulated concrete blocks with slotted polystyrene limit heat loss in the winter and minimize heat gain in the summer [42].

### Limitations

Although the study findings are useful and applicable to the South African context, there were several limitations. One limitation of this study was that measurements were not taken in all months of the year; a few weeks within the spring season were missing, and there were no measurements across the summer season, which would indicate greater disparities in ventilation and air conditioning systems between rural and urban classrooms. In addition, measurements were taken from mid-Autumn, so the full picture of seasonal temperature disparities has not been accounted for. Another limitation is that the geographic orientation of the classrooms was not captured (e.g., east-facing versus north-facing), which could explain cooling effects in some of the buildings [43]. Thermal comfort standards may be influenced by building characteristics, geographical factors, and individual factors. The lack of tree shade in the rural classrooms contributed to the higher measured temperatures, which would be a greater concern in the summer season, which was not included in this study.

We also did not measure insulation presence/absence, which would likely impact temperatures inside classrooms. This study was conducted at urban and rural schools in South Africa, and there are many other climatic zones to be explored, including mountains, grasslands, bushveld, and coastal regions. Previous studies on the effects of heat on the thermal comfort of scholars have been undertaken. Absenteeism, academic achievement, headaches, tiredness, and dehydration, as well as other indicators of heat stress, should be included in future studies. The rate at which scholars are affected by inadequate thermal comfort in different climatic zones may provide further motivation for climate-proofing of classrooms in South Africa.

## Conclusion

This study demonstrates clear urban-rural disparities in classroom thermal comfort in South Africa, with rural classrooms in Giyani experiencing substantially higher and more variable indoor temperatures due to poorer building insulation, absent or damaged ceilings, and limited effectiveness of low-energy cooling measures such as fans. The findings underscore the importance of targeted, context-specific interventions, particularly the improvement of basic building integrity, enhancement of ventilation, and investment in ecological shading through trees, as priority adaptation measures for rural schools. Within the context of a just energy transition, these results highlight the need for equitable investment in climate-resilient educational infrastructure, ensuring that energy-efficient cooling solutions and improved building design are directed toward the most heat-vulnerable and under-resourced school environments. The study's implications extend beyond South Africa, as it calls for a more equitable approach to educational infrastructure development in LMICs, emphasizing energy-efficient solutions like solar air-conditioning and green building designs. Addressing these disparities can contribute to reducing the adverse effects of climate change on vulnerable populations, particularly in resource-constrained regions.

### What is known about this topic

- High indoor temperatures in classrooms, particularly in low-income settings, can negatively impact children’s learning, attention, and overall comfort;
- Building characteristics contribute to elevated indoor temperatures.

### What this study adds

- Empirical evidence of substantial differences in indoor thermal conditions between urban and rural South African classrooms, highlighting the influence of classroom construction and environmental exposure;
- Identification of specific classroom features such as intact ceilings and fan cooling that lower indoor heat provides practical insights for climate-smart school infrastructure policies.

## Annex

**Annex 1**: supplementary materials (PDF - 303KB)
